# DNA Gyrase and Topoisomerase IV Mutations and their effect on Quinolones Resistant *Proteus mirabilis* among UTIs Patients

**DOI:** 10.12669/pjms.36.6.2207

**Published:** 2020

**Authors:** Randa H Abdelkreem, Amjad M Yousuf, Miskelyemen A. Elmekki, Mogahid M Elhassan

**Affiliations:** 1 Randa H Abdelkreem Dept. of Microbiology, College of Medical Laboratory Science, Shendi University, Shendi, Sudan; 2 Amjad M Yousuf, Dept. of Medical Laboratory Technology, College of Applied Medical Sciences, Taibah University, Al-Madinah Al-Monawwarah, Saudi Arabia; 3 Miskelyemen A. Elmekki, Dept. of Medical Laboratory Technology, College of Applied Medical Sciences, Taibah University, Al-Madinah Al-Monawwarah, Saudi Arabia; 4 Mogahid M Elhassan, Dept. of Medical Laboratory Technology, College of Applied Medical Sciences, Taibah University, Al-Madinah Al-Monawwarah, Saudi Arabia

**Keywords:** Ciprofloxacin resistance, *Proteus mirabilis*, Sudan

## Abstract

**Objective:**

This study aimed to highlight the importance of mutations within *Proteus mirabilis* genome that are related to fluoroquinolone resistance.

**Methods:**

This is a cross sectional study performed in different teaching hospitals in Khartoum State from June 2016 to May 2017. A total of (120) *P mirabilis* isolates from patients with symptoms of UTIs attending different hospitals in Khartoum State were examined. First, modified Kurby Bauer method was performed for phenotypical detection of resistant isolates. Then polymerase chain reaction-restriction fragment length polymorphism (PCR-RFLP) followed by sequencing were applied for detection of mutations in *GyrA*, *GyrB*, *ParC* and *ParE* genes of isolates.

**Results:**

*P. mirabilis* showed 30% resistance to ciprofloxacin. All samples revealed mutation at (serine 83) of *GyrA* and (serine 84) of *ParC* by *Hinf1* restriction endonuclease digestion. Sequencing was performed for 12 samples. For each gene, two resistant and one susceptible strains were randomly selected. The mutations associated with ciprofloxacin resistant *P. mirabilis* were as follows; (1/3) *GyrA* (Ser 83 to Ile) and (2/3) *ParC* (Ser 81 to Ile). Also it revealed silent mutations at codons of *GyrB* 474 leucine (3/3), 585 valine (2/3), 612 histidine (1/3) and 639 asparagine (1/3) and *ParE* 469 isoleucine (2/3), 531 aspartic (2/3) and 533 glycine (1/3).

**Conclusions:**

Ciprofloxacin resistance in *P. mirabilis* could be monitored through detection of mutations within DNA gyrase (encoded by gyrA and gyrB) and topoisomerase IV (encoded by parC and parE).

## INTRODUCTION

Proteus mirabilis is a small gram-negative bacilli and a facultative anaerobe, it ferments maltose, but not lactose. Moreover, Proteus mirabilis is one of the common causes of urinary tract infections (UTIs) among Enterobacteriaceae.^1^

Ciprofloxacin is a recommended drug for the treatment of UTIs.^2^ Though wild-type strains of P. mirabilis are usually susceptible to fluoroquinolones^2^,^3^ but a progressive increase in fluoroquinolone resistance has been seen in the clinical isolates of the bacterium recently.^3^,^4^

The basic mechanisms of quinolone resistance are represented by the changes in the active sites of the target enzymes DNA gyrase and topoisomerase IV. The degree of resistance of different regions (QRDRs) encoded by gyrA and parC gene mutations have been described in several studies.^5^ In Sudan, a recent study that analyzed the antimicrobial susceptibility patterns of several species of Gram-negative bacteria, including *P mirabilis*, to four different groups of antibiotics showed that (22.3%) of the isolates were resistant to three or more classes of antibiotics, including cephalosporins, β-lactam–β-lactamase inhibitor, quinolones, aminoglycosides and carbapenems.^6^

## METHODS

The study was carried out using 120 *Proteus mirabilis* urinary isolates collected from different hospitals in Khartoum State. The isolates were collected during the period from June 2016 to May 2017. This study obtained ethical approval number (MLT 711/2016) from the ethical committee of SUST.

### Bacteriology

Urine samples were cultured and P.mirabilis was isolated and identified by the conventional standard methods. All the grown isolates were tested for their ciprofloxacin resistance *in vitro* by the Kirby-Baur disk diffusion method against ciprofloxacin (CIP) (5 μg/ml).^7^

### Polymerase Chain Reaction

### DNA Extraction

DNA of *Proteus mirabilis* was isolated from overnight growth on nutrient agar. For each isolate, several colonies of pure culture were suspended in (500 μL) of sterile deionized water in 1.5 ml eppendorf tube for each isolate, and boiled for (10 minutes). Then tubes were centrifuged at (14000 g) for (10 minutes) using a microcentrifuge and supernatant was stored at (-20°C) as a template DNA stock.^8^ The purity of the extracted DNA was determined by running the DNA sample on (2%) agarose gel.^9^

### Primer Design

Degenerate oligonucleotide primers (Table-I) from conserved regions of the *GyrA*, *GyrB*, *ParC* and *ParE* genes were designed by primer3plus (www.bioinformatics.nl/primer3plus) from *Proteus mirabilis* HI4320 DNA sequences in the Gen Bank database (NCBI) and were synthesized by Macrogen (South Korea).

**Table-I:** Primers used for detection of virulence genes in *Proteus mirabilis* strains.

Primer	primers Sequence	Product size bp
*Gyr A*	F 5‘- AGCGACATTGCCAGAGAAAT -3‘ R 5‘- CACCGACTGCATCACGTTT -3‘	937
*Gyr B*	F 5‘- GGCAAAACAAGGGCGTAA-3‘ R 5‘- GCCCCTTCTTCAATCAGGTT-3‘	822
*Par C*	F 5‘- CAGCGTCGTATCGTCTATGC-3‘ R 5‘-CGGCGTAATACTTTTTCTAAGC-3‘	992
*Par E*	F 5‘- GGAAGGAGGCGATTTACTCA-3‘ R 5‘-GGATCAAGCGTTGTCTCACG-3‘	972

### Amplification of GyrA, GyrB, ParC and ParE Genes

DNA amplification was done using Maxime PCR Premix kit (*I*-Taq) (iNtRON, Korea) which is a lyophilized master mix. The PCR assay was carried out in a total volume of (20 μL) of mixture containing (0.5 μL) of each of the virulence gene-specific primers (1 μL total volume for forward and reverse primer in each case), (2 μL) of template DNA and (17 μL) of water for injection (WFI). The amplification was done using (CLASSIC K960 China thermal cycler).

### Restriction Fragment Length Polymorphism (RFLP-PCR)

The PCR product was digested with *HinfI* restriction enzyme (CutSmart™, New England Biolabs, Inc) and endonuclease digestion was performed as recommended by the manufacturer to detect *GyrA* (ser 83) and *ParC* (ser 81) mutations.

### Sequencing of the Target Genes

Three products were selected randomly to detect *GyrA, GyrB*, *ParC* and *ParE*, Sequencing was performed in both directions with the same set of primers used for the PCR by Sanger dideoxy chain termination method.

### Data and Genetic Analysis

The data was analyzed using statistical software package (SPSS - version 20). The sequences were checked for similarity with reference genes using NCBI’s BLAST (http://www.ncbi.nlm.nih.gov/blast). The sequences were translated into amino acid codons using Expasy translation tool. The protein sequences were then checked for similarity in BLAST.

## RESULTS

### Bacteriological Findings

### Culture

The identification scheme confirmed that (120) of the isolates belonged to the species *P. mirabilis*.

### Disk Diffusion Method

The results of modified Kirby-Bauer method showed that *P. mirabilis* reflected relatively decreased sensitivity to ciprofloxacin as only 84 (70%) of the isolates were sensitive while 36 (30%) were resistant, with a statistically significant difference (p=0.000).

### Polymerase Chain Reaction (PCR)

### PCR for the amplification of GyrA, GyrB, ParC and ParE Genes

**Fig.1:**
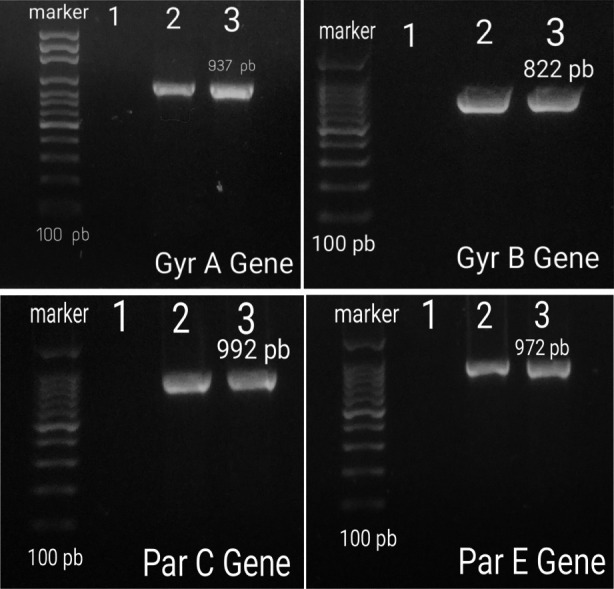
PCR products of GyrA, GyrB, ParC and ParE gene products on 2% agarose gel; lane 1: Negative control, lanes 2 and 3: PCR products.

Degenerate oligonucleotide primers from conserved regions of the *GyrA*, *GyrB*, *ParC* and *ParE* genes were designed from alignments of known DNA sequences in the Gen Bank database (NCBI). PCR amplification targeted *GyrA* gene product (937 bp) which encoded (312) amino acids, *GyrB* gene product (822bp) which encoded (274) amino acids, *ParC* gene product (992bp) which encoded (230) amino acid and *ParE* gene product (972bp) which encoded (324) amino acids on *P. mirabilis* as seen on 2% agarose gel (Fig.1).

### Hinf Digestion of GyrA and ParC Genes

**Fig.2:**
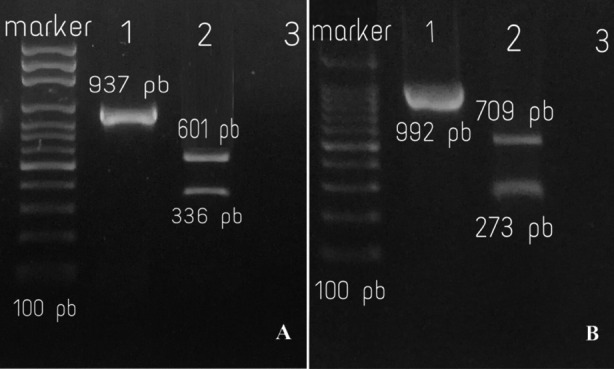
PCR products of GyrA and ParC were digested with HinfI and separated by 2% agarose gel.A (GyrA); lane 1: non-digested products (937 bp), lane 2: HifI-digested product (601 and 336 bp) lane 3: negative control and B (ParC); lane 1: non-digested products (992 bp), lane 2: HifI-digested product (709 and 273 bp) lane 3: negative control.

Quick screening of the (120) isolates of *P.mirabilis* to detect the mutations at the codon (83 ser) of the *GyrA* gene and (84 ser) of *ParC* was done by *Hinf1* restriction endonuclease digestion. PCR amplified *GyrA* gene product (937 bp) of all the isolates (mutant 83ser: two bands 601 and 336 bp, non-mutant 83 ser: three bands 601, 200 and 136 bp), and *ParC* product (992 bp) of all the isolates (mutant 84ser: two bands 709 and 273 bp, non-mutant 84 ser: three bands 656, 273 and 53 bp) as seen on the agarose gel (Fig.2) all samples showed mutations at (serine 83) of *GyrA* and (serine 84) of *ParC*.

### Sequencing of GyrA, GyrB, ParC and ParE Genes

**Fig.3:**
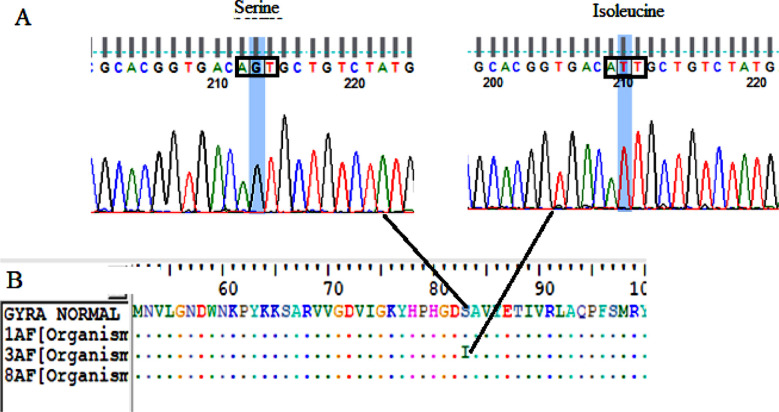
Line (A) Chromatograms of Sanger DNA sequencing of GyrA changed from G to T which change serine to isoleucine, line (B) GyrA amino acid changed codon 83 serine to isoleucine (AGT- ATT). Analyses was done by BioEdit alignment editor v7.2.5

**Fig.4:**
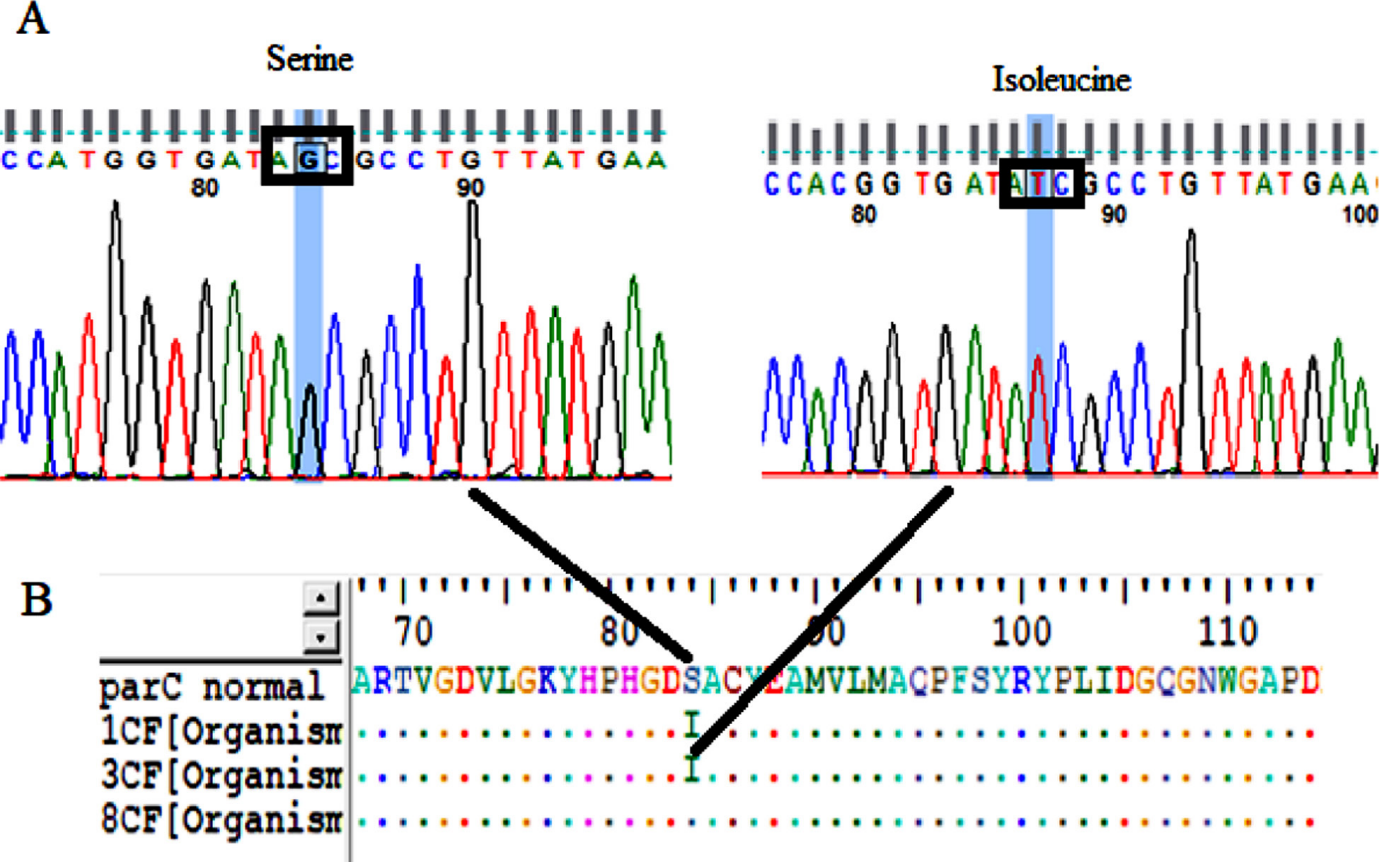
Line (A) Chromatograms of Sanger DNA sequencing of ParC serine 84 changed from G to T isoleucine, line (B) ParC amino acid changed codon 84 serine to isoleucine (AGC- ATC). Analyses was done by BioEdit alignment editor v7.2.5

Sequencing of all QRDR *P. mirabilis*, performed by alignment with reference strain *P. mirabilis* HI4320 in GenBank database NCBI by nucleotide blast revealed:

- Mutations at (codon 83) of *gyrA* possessed serine to isoleucine substitution (G 248 T) this was observed in one strain (33.3%) (Table-II and Fig.3).
- Mutations at codon 84 and silent mutation at codon 81 and 116 of *ParC*. Serine 84 to isoleucine (66.6%) at resistant strains (1C and 3C) substitution (G 251 T), codon 81 histidine (33.3%) in sensitive strain (8C) substitution (C 243 T) and codon 116 proline (33.3%) in resistant strain (3C) substitution (A 348 T) (Table-II and Fig.4).
- Silent mutations at codons 474, 585, 612 and 639 of *GyrB*, Codon 474 leucine in all strains (100%) including sensitive strain (8B) substitution (A 1422 G), codon 585 valine (66.6%) in resistant strains (1B and 3B) substitution (T1 755 C), codon 612 histidine (33.3%) in sensitive strain (8B) substitution (C1836T) and codon 639 asparagine (33.3%) in sensitive strain (8B) substitution (T1917C) (Table-II).
- Silent mutations at codons (469, 531 and 533) of *ParE*, Codon (469) isolusine (66.6%) in resistant stain (3E) and sensitive strain (8E) substitution (C 1407 T), codon (531) aspartic acid (66.6%) in resistant stain (3E) and sensitive strain (8E) substitution (C1593T) and codon (533) glycine (33.3%) in sensitive strain (8E) substitution (1 599 A) (Table-II).

**Table-II:** Accession numbers, ciprofloxacin susceptibility and QRDR mutations of * Proteus mirabilis* isolates.

Sample	accession numbers	Ciprofloxacin susceptibility	Target gene	Amino acid change	

				Amino acid	Nucleotide
1A	MH310924	Resistance	*GyrA*	-	-
3A	MH310925	Resistance	*GyrA*	Ser 83 Ile	AGT-ATT
8A	MH310926	Sensitive	*GyrA*	-	-
1B	MH310921	Resistance	*GyrB*	Lus 474 Lus	TTA –TTG
				Val 585 Val	GTT –GTC
3B	MH310922	Resistance	*GyrB*	Lus 474 Lus	TTA –TTG
				Val 585 Val	GTT –GTC
8B	MH310923	Sensitive	*GyrB*	Lus 474 Lus	TTA –TTG
				His 612 His	CAC-CAT
				Asn 639 Asn	AAT AAC
1C	MH310927	Resistance	*ParC*	Ser 84 Ile	AGC-GTC
3C	MH310928	Resistance	*ParC*	Ser 84 Ile	AGC-GTC
				Pro 116 Pro	CCA CCT
8C	MH310929	Sensitive	*ParC*	His 81 His	CAC CAT
1E	MH310930	Resistance	*ParE*	-	-
3E	MH310931	Resistance	*ParE*	Ile 469 Ile	ATC –ATT
				Asp 531 Asp	GAC-GAT
8E	MH310932	Sensitive	*ParE*	Ile 469 Ile	ATC –ATT
				Asp 531 Asp	GAC-GAT
				Glu 533 Glu	GGT-GGA

## DISCUSSION

During the last decade, an increase in the incidence of fluoroquinolones resistance was reported among aerobic Gram negative bacilli. However, full recognition of reports regarding the emergence of fluoroquinolone resistant *P. mirabilis* strains is still under investigation. Moreover, the genus *Proteus* is isolated from patients, especially from those with UTIs.^3^

The results obtained from this study showed that (30%) of *P. mirabilis* isolates were resistant to ciprofloxacin, this finding agrees with Rajivgandhi *et al*. from India^10^ and Kyung *et al*. from Korea^11^ who detected (30%, 28%, 27%) of resistance *of P. mirabilis* resistance to ciprofloxacin, respectively. Different studies revealed different findings; in Sudan, Amir *et al*. found no resistance to ciprofloxacin in *P. mirabilis*.^12^ On the other hand, in Japan, lower percentage of resistance was found (16%)^13^ while higher percentage was observed in Poland and in Taiwan; (40 % and 68.7%), respectively.^14^,^15^

Generally, the possible reasons behind the resistance to ciprofloxacin in Sudan may be the fact that this antibiotic have been in use for a long period and must have been abused, leading most properly to a change in the genome of the bacteria, making the target site of the antibiotic action inaccessible. Important mechanisms of bacterial resistance to quinolone are the genetic mutations in the subunits *GyrA* and *ParC* of DNA gyrase and topoisomerase IV enzymes, as well as the subunits *GyrB* and *ParE*, which are also components of the target enzymes.^13^

*P. mirabilis* always mutate in *GyrB* (Ser 464 to Tyr or Phe), as mentioned by Saito *et al*.^13^ This amino acid is not present in *P. mirabilis GyrB* sequence of clinical isolates but revealed silent mutations in the following codons; (474) leucine, (585) valine, (612) histidine and (639) asparagine. Also *ParE* gene always mutates in (Val 364 to Iso) in *P. mirabilis*,^13^, Thr-86-Ile, mutation from GyrA was the most common in Campylobacter jejuni^16^. In this study, sequence analysis of *ParE* gene fragments from the clinical isolates revealed silent mutations in codons (469) isoleucine, (531) aspartic and (533) glycine. However, no mutations were detected in the corresponding region of *pare* neither in quinolone resistant nor in sensitive *P. mirabilis* isolates of this study. However, it is well known that *ParE* does not have an essential role in fluoroquinolone resistance among *P. mirabilis* as suggested previously.^13^

In this study, ciprofloxacin resistant *P. mirabilis* possessed mutations in *GyrA* (Ser 83 to Ile). This amino acid change is identical to those previously reported for fluoroquinolone resistance^13^ where *P. mirabilis* mutations in *GyrA* (Ser 83 to Arg or Ile) was proved. Other studies showed different mutations in *GyrA* with other bacteria; (Ser 80 to Leu 86) of *Capnocytophaga* spp.^17^, (Ser 83 to Leu) of *E. coli*^18^ and (Ser 83 to Phe) of *M. bovis*.^19^

*P. mirabilis* sequencing of *ParC* showed mutation in (Ser 84 to Ile) in this study. This result is in agreement with many researches who proved that *Proteus mirabilis* always mutated in GyrA (E87) and ParE (D420) for fluoroquinolone resistance.^3^ Also *Edwardsiella tarda*, another Gram negative bacillus, was found mutant in (Ser 84 to Ile) of *ParC* which is associated with fluoroquinolone resistance.^5^

In gram negative bacilli, fluoroquinolone resistance is mostly attributed to the antibiotic targets DNA gyrase and DNA topoisomerase IV structure change as the most significant mechanisms.^20^ In *E. coli*, resistance to ciprofloxacin may be obtained by more than two mutations in both *GyrA* and *ParC* genes.^13^,^20^ In this study however, only one or double mutation are enough for ciprofloxacin resistance in P. *mirabilis*.

In *P. mirabilis*, decreased susceptibility to fluoroquinolone is caused by mutations at residues (Ser 80 and Glu 84) of *ParC* of topoisomerase IV, a target of quinolones.^13^ Although both *ParC* and *GyrA* mutations are needed for acquisition of quinolones resistance^13^, in this study, one of the clinical isolates was found to have mutations only in *ParC* but not *GyrA*, which suggest that *ParC is* as important as *GyrA* in decreasing susceptibility to fluoroquinolones in *P. mirabilis*.

In contrast to the case of *Acinetobacter baumannii*, where silent mutation in QRDR regions were reported to be sufficient for fluoroquinolone resistance^21^, in *P. mirabilis*, sensitive strains in this study were found to possess silent mutation in *GyrB*, *ParC* and *ParE*.

Direct Hinf1 digestion of PCR product have been used by many researchers to screen GyrA and *ParC* genes mutations in different bacteria; *S. pneumoniae* at positions serine (83) of *GyrA* and serine (79) of *ParC*^22^, *A. baumannii* at positions *gyrA* (codons 83 and 87) and *parC* (codons 80 and 84)^23^ and *N. gonorrhoeae* at positions Ser (91) of *GyrA*^24^ were all found significantly associated with ciprofloxacin resistance.

In this study, direct *Hinf1* digestion of PCR amplicons have been used to screen *GyrA* and *ParC* mutations in *P. mirabilis*. Mutations at codon (83) of the *GyrA* gene and (84) of *ParC* gene result in the loss of natural *Hinf*1 site as identified. The results indicated that all resistant isolates mutated at serine (83) of *GyrA* and serine (84) of *ParC*. When dealing with sequencing, two out of the three sequenced *GyrA* appeared as non-mutated at Ser (83) while one sample of the other three sequenced *ParC* resulted as free from any mutations at ser (84). These findings may be attributed to the fact that serine (83) of *GyrA* and serine (84) of *ParC* in *Proteus mirabilis* consist of (AGC) which is almost different from serine in other bacteria (TCC). Thus the loss of natural *Hinf*1 site (5’…GANTC…3’) will result.

In Sudan, many studies have been conducted to analyze antibiotic resistance in Gram-negative and Gram-positive bacteria isolated from different clinical specimens of both humans and animals. A wide range of resistance was detected which represent an alarm to the health authority of the country to take an action in order to control this phenomena.^25^

### Limitations of the study

This research received no special fund, thus sequencing was performed to limited number of the isolates with random selection. Moreover, sample collection was limited only to the central teaching hospitals and the rural and terminal medical centers were not reachable.

## CONCLUSION

In conclusion, direct *Hinf1* digestion of PCR amplicons is not suitable to screen serine (83) of *GyrA* and serine (84) of *ParC* mutations in *P. mirabilis*. In addition *, Proteus mirabilis ParC* gene is as important as *GyrA* gene to cause ciprofloxacin resistance, with only one or two mutations in both *GyrA* and *ParC* genes of *Proteus mirabilis* being enough to obtain resistance to ciprofloxacin. Moreover, the study drew the attention of the clinicians to consider the percentage of resistance to Quinolones in order to try other options of treatment, that means drug susceptibility testing should be adopted to all patients of similar infections before starting a specific treatment.

### Authors Contribution:

**RA: S**pecimen collection and molecular biology work.

**ME: A**pproval of the proposal and general supervision, orientation and arrangement.

**ME & AY: D**ata analysis and manuscript writing and revision.

**ME:** Is responsible for the integrity of the entire work. All authors have contributed to this manuscript.
